# Case Report: Efficacy, safety, and favorable long-term outcome of early treatment with IL-1 inhibitors in a patient with chronic infantile neurological cutaneous articular (CINCA) syndrome caused by NLRP3 mosaicism

**DOI:** 10.3389/fped.2024.1379616

**Published:** 2024-04-24

**Authors:** Giorgio Costagliola, Sofia D’Elios, Susanna Cappelli, Francesco Massei, Giulia Maestrini, Alessandra Beni, Diego Peroni, Rita Consolini

**Affiliations:** ^1^ Section of Pediatric Hematology and Oncology, Azienda Ospedaliero Universitaria Pisana, Pisa, Italy; ^2^ Section of Clinical and Laboratory Immunology, Division of Pediatrics, Department of Clinical and Experimental Medicine, University of Pisa, Pisa, Italy; ^3^ Division of Pediatrics, Department of Clinical and Experimental Medicine, University of Pisa, Pisa, Italy

**Keywords:** anakinra, autoinflammatory disorders, canakinumab, chronic infantile neurological cutaneous articular syndrome, cryopyrin-associated syndromes (CAPS), inflammasome

## Abstract

Chronic infantile neurological cutaneous articular (CINCA) syndrome is an autoinflammatory disease encompassed in the group of cryopyrin-associated periodic syndromes (CAPS). Patients suffering from CINCA have an elevated risk of developing chronic sequelae, including deforming arthropathy, chronic meningitis, neurodevelopmental delay, and neurosensorial hearing loss. The diagnosis of CINCA presents several difficulties, as the clinical phenotype could be difficult to recognize, and almost half of the patients have negative genetic testing. In this paper, we describe the case of a patient presenting with the typical phenotype of neonatal-onset CINCA who resulted negative for NLRP3 mutations. Based on the clinical judgment, the patient underwent treatment with anti-interleukin-1 (IL-1) agents (anakinra and, later, canakinumab) resulting in a complete clinical and laboratory response that allowed confirmation of the diagnosis. Additional genetic investigations performed after the introduction of anti-IL-1 therapy revealed a pathogenic mosaicism in the NLRP3 gene. After a 12-year follow-up, the patient has not experienced chronic complications. Although genetics is rapidly progressing, this case highlights the importance of early diagnosis of CINCA patients when the clinical and laboratory picture is highly suggestive in order to start the appropriate anti-cytokine treatment even in the absence of a genetic confirmation.

## Introduction

Cryopyrin-associated syndromes (CAPS) are a group of diseases featured by a dysregulated inflammatory response, which characterizes autoinflammatory diseases (1). The pathologic hallmark of CAPS relies on activating mutations of the NLRP3 (CIAS1) gene, encoding for cryopyrin. Cryopyrin is a central component of the NLRP3 inflammasome, a complex of intracellular proteins (2), which results in the induction of caspase-1 and release of IL-1β (3). The unrestricted activation of caspase-1 and deregulated secretion of IL-1β lead to systemic and multiorgan sterile inflammation. As a consequence of its pivotal role in inducing a large spectrum of secondary inflammatory mediators, excessive IL-1 activity causes a broad variety of organ dysfunctions. IL-1 blockade represents the therapeutic strategy to hinder the disease progression and IL1-inhibitors are presently approved for the treatment of CAPS. Notably, almost half of the described patients do not show the NLRP3 mutations, thus evidencing that the CAPS genetic background is not completely elucidated (4, 5). Historically, CAPS were classified into three main disorders, depending on disease severity: familial cold-associated syndrome (FCAS), Muckle-Wells syndrome, and chronic infantile neurological cutaneous articular (CINCA) syndrome (6). The clinical spectrum ranges from patients with self-resolving disease to those developing chronic complications, including sensorial hearing loss and neurodevelopmental delay (1). CINCA syndrome represents the most severe phenotype of the CAPS spectrum, being associated with neonatal onset and long-term complications (6). Herein we describe the case of a patient diagnosed with CINCA syndrome caused by NLRP3 mosaicism during the second year of life and treated with IL-1 inhibitors, analyzing the outcome after a 12-year follow-up.

## Case presentation

We describe the case of a female born at term from an uneventful pregnancy, with a positive familial history for autoimmune disorders (mother with vitiligo, grandmother suffering from rheumatoid arthritis) but negative for autoinflammatory disorders. Since the first days of life, the patient presented episodes of diffuse, severe, and drug-resistant urticaria, in the absence of evident physical, infectious, or other identifiable triggers. From the fifth month of life, she presented multiple episodes of fever, recurring every 40 days, with a duration ranging from 7 to 10 days, in the absence of specific clinical manifestations. The episodes were self-limiting and unresponsive to antibiotic therapy.

The patient underwent numerous unsuccessful specialistic consultations, which also included an allergology work-up with skin prick tests that ruled out food allergies.

**Figure 1:**
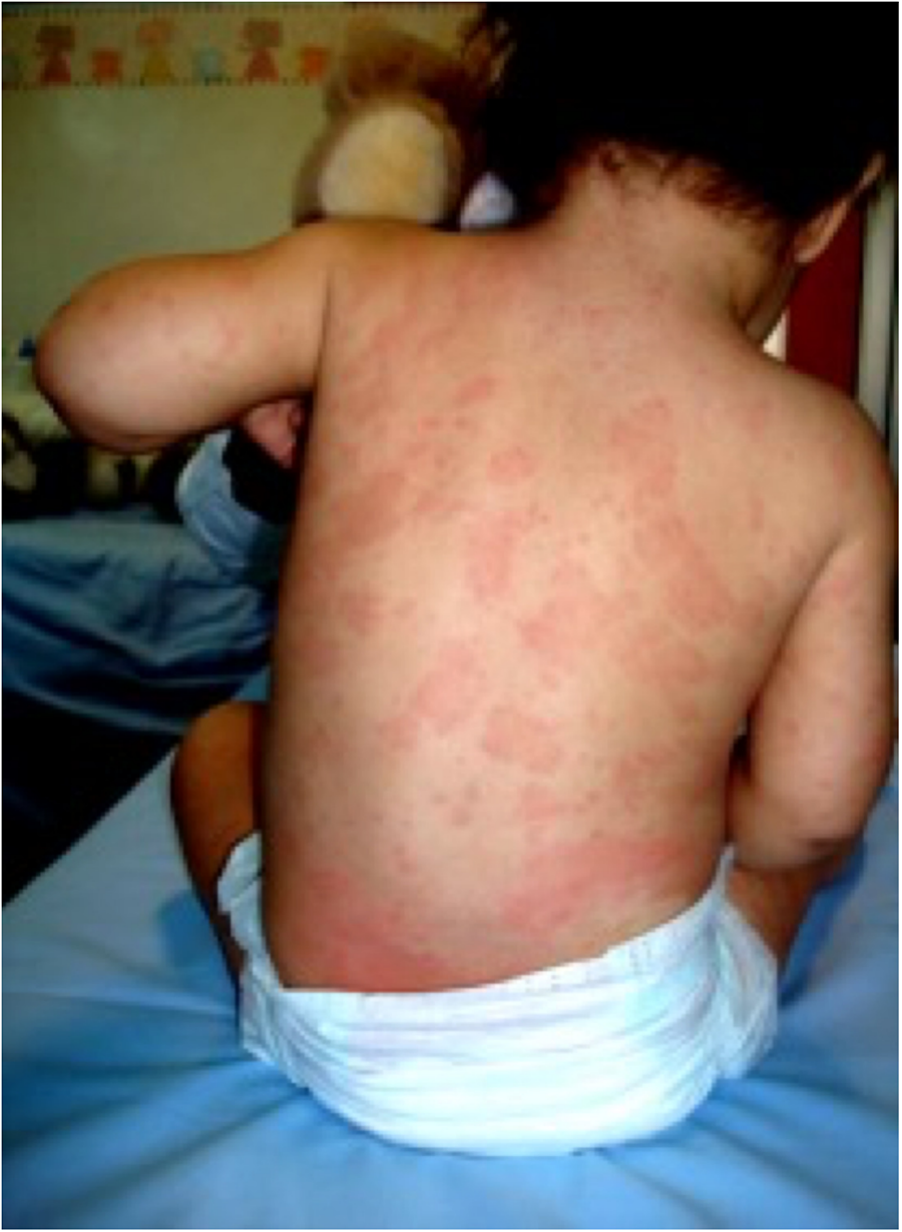
Typical urticarial rash in our patient.

At the age of 18 months, for the persistence of the clinical picture, the patient was referred to our rheumatology clinic. At physical examination, she presented a diffuse, non-itchy urticarial rash (Figure 1), facies featured by frontal bossing and saddle nose, and mild deformities of the fingers, which appeared slightly enlarged. Enlarged lymph nodes were appreciable in cervical, axillary, and inguinal stations. The remaining physical assessment did not reveal pathological findings, and neurological assessment was normal for her age. Laboratory investigations showed a raise in inflammatory markers [erythrocyte sedimentation rate (ESR) 110 mm/h, C-reactive protein (CRP) 3.65 mg/dl, serum amyloid protein (SAP) 64.6 ug/ml] with leukocytosis (white blood cells 22,790/mmc, neutrophils 60%, lymphocytes 34%), microcytic anemia (8.5 g/dl with mean globular volume 58 fl), mild elevation of IgG immunoglobulins for her age (1,350 mg/dl), and normal thyroid function. The infectious screening was negative, and the analysis of lymphocyte subpopulation evidenced an increase of CD19+ B cells (47.1%) in the absence of a significant T-cell defect. Phagocyte respiratory burst function was tested and the results were normal, and an extended autoantibody screening was negative. Based on the clinical and laboratory features (recurrent fever, urticaria, skeletal abnormalities, and elevated inflammatory markers) the prospect of CAPS was posed. Particularly, the neonatal onset, the chronic course of urticaria, and the typical facies raised the clinical suspicion of CINCA syndrome. Therefore, the patient underwent genetic testing for NLRP3 mutation with Sanger sequencing. Audiological evaluation with auditory brainstem response was performed and did not evidence any pathological findings. Additionally, the patient underwent neuropediatric and ophthalmological assessment, which returned normal results, and a leg ultrasound that was negative for patellar dysplasia. Following the clinical suspect, at 20 months of age the patient started a treatment of anakinra, administered subcutaneously at a dosage of 1 mg/kg daily. The treatment resulted in a dramatic resolution of the febrile pattern and cutaneous lesions 24 h after the administration. Similarly, inflammatory markers showed a remarkable reduction the day after and progressive normalization. Despite the negativity of the genetic testing for NLRP3 mutations, the clinical and laboratory picture, together with the clear response to the therapeutic trial with IL-1 blockade, confirmed the clinical diagnosis of CINCA (7, 8). In the months following the introduction of anakinra, amplicon-based NLRP3 deep sequencing was performed, evidencing a 10% mosaicism (R260P) in the NLRP3 gene in our patient. This genetic variant has been previously reported to be associated with severe CINCA syndrome evolving with persistent arthropathy and articular deformities (9).

From the initiation of anakinra, the patient did not experience other CAPS-associated febrile episodes or urticaria. The drug has been well tolerated, although the patient experienced sporadic oral apthosis and headache. After two years of treatment, the child displayed an elevation of inflammatory markers (ESR 50 mm/h, CRP 1.67 mg/dl, SAP 57.9 μg/ml) in the absence of specific clinical manifestations. Therefore, anakinra was withdrawn and replaced with canakinumab 6 mg/kg subcutaneously every 8 weeks. This therapeutic approach was effective in improving the patient's quality of life, maintaining the clinical remission, and reducing the inflammatory markers, which remained negative during the following periodic laboratory assessments. After the introduction of canakinumab, the patient did not experience adverse reactions, and the episodes of apthosis and headache were no longer reported. Additionally, the patient showed satisfactory neurological development, with regular school attendance until the age of 14 years, and good socialization. Twelve years after the introduction of anti-IL-1 blockade, the patient is still in clinical remission; she presents a normal auxological development and an adequate neurological development, in view of the family's low socio-cultural level, and has negative inflammatory markers. Additionally, she did not develop chronic deforming arthropathy, and the periodic audiological and ophthalmological surveillance did not evidence the typical chronic complications associated with CINCA. At the last follow-up visit, her disease is inactive, and the autoinflammatory disease damage index (ADDI) (10) is 2 since the patient has only presented pubertal delay and amenorrhea.

## Discussion

The diagnosis of CAPS often presents several difficulties, and the diagnostic delay can be consistent. Despite the early disease onset, in large case series, the mean diagnostic delay is 14 years (4, 11). In this regard, the absence of specific serum biomarkers and the low sensibility of the genetic testing (high percentage of patients without NLRP3 mutations) explain the importance of the physician's awareness.

In the described case, although the patient presented clinical and laboratory features suggestive of CAPS (and, specifically, CINCA syndrome), the diagnosis was complicated by the incomplete disease expression at disease onset. Specifically, the absence of hearing impairment, which is one of the most relevant warning signs of CINCA (11), could partly explain the initial diagnostic delay.

The neonatal onset, together with the skeletal abnormalities, were elements that identified the patient as high risk for the development of chronic sequelae. Indeed, chronic inflammation, if not adequately controlled, is responsible for non-reversible complications involving the joints, the ear, and the central nervous system (6).

From literature data, it emerges that up to 50% of the patients diagnosed with CINCA develop deforming arthropathy, beginning during childhood, and 40%–60% of the patients show sensorineural hearing loss during follow-up. Additionally, chronic meningitis is reported in up to 20% of the patients in the EUROFEVER registry, and patients can also show uveitis and optic disc abnormalities during the disease course (12).

Currently, the diagnostic awareness of CAPS is increasing, and there are reports of newborns and infants successfully diagnosed and treated (13–15). However, the rate of misdiagnosis before the results of genetic testing could be considerable (14). The diagnostic process in newborns presents several difficulties, as the disease often presents only with the cutaneous findings and the elevation of inflammatory markers, while other disease-associated signs develop in later stages (16).

In patients with CINCA, treatment is started before genetic confirmation only in a small percentage of the reported cases (17). Therefore, patients without a detectable mutation may show a significantly higher time to diagnosis and delayed treatment, thus favoring the development of the chronic sequelae.

The favorable long-term outcome after early treatment of the case described suggests that, in the case of a patient with highly suggestive clinical and laboratory picture, early treatment with IL-1 inhibitors is recommended, without waiting for the result of the genetic analysis. This is of particular relevance for patients with moderate or severe phenotypes. The use of anakinra, as a diagnostic as well as a treatment tool, due to its short half-life, rapid onset of action, and excellent safety profile, may be indicated for patients with undefined signs or symptoms of autoinflammation (18).

In the case of negative genetic testing, the search for mosaicisms in the NLRP3 gene is of extreme utility to confirm the clinical suspect, as previous studies identified that more than 60% of patients with CINCA syndrome and negative genetic investigations show variable degrees of mosaicism (17). Clear genotype-phenotype correlations in patients with mosaicism are lacking (19), and the degree of disease severity and long-term consequences in these patients have to be further explored. With specific regard to the presented case, although the genetic variant carried by our patient has been previously described in association with a severe clinical phenotype, its impact in the context of mosaicism is not univocally defined. This report could suggest that, if adequately and promptly treated, patients with NLRP3 mosaicism could be less susceptible to the development of chronic sequelae (20).

However, while benefitting from duration of the follow-up, this paper represents a single observation, and there is a need for long-term follow-up data from patients with mosaicisms to better characterize their outcome.

Hopefully, the use of specific next-generation sequencing (NGS) panels for autoinflammatory disorders, together with large genetic data networks, will help to better understand the complex genetic background of CAPS and to provide specific correlations with the clinical phenotype to finally guide the treatment and follow-up approach. Moreover, the increasing awareness of the possibility to diagnose CAPS in early life could help in providing timely treatment and reducing the development of chronic sequelae.

## Data Availability

The original contributions presented in the study are included in the article/Supplementary Material, further inquiries can be directed to the corresponding author.
